# Protective Effects of Keratinocyte-Derived GCSF and CCL20 on UVB-Induced Melanocyte Damage

**DOI:** 10.3390/cells13191661

**Published:** 2024-10-08

**Authors:** Saowanee Jeayeng, Malinee Saelim, Phetthinee Muanjumpon, Pongsakorn Buraphat, Potjanee Kanchanapiboon, Somponnat Sampattavanich, Uraiwan Panich

**Affiliations:** 1 Department of Medical Science, School of Medicine, Walailak University, Nakhon Si Thammarat 80160, Thailand; saowanee.j10@gmail.com; 2 Research Center in Tropical Pathobiology, Walailak University, Nakhon Si Thammarat 80161, Thailand; 3 Department of Pharmacology, Faculty of Medicine Siriraj Hospital, Mahidol University, Bangkok 10700, Thailand; malinee_saelim@hotmail.co.th (M.S.); phetthinee.m@gmail.com (P.M.); 4 Department of Medicine, Faculty of Medicine Siriraj Hospital, Mahidol University, Bangkok 10700, Thailand; 5 Division of Nuclear Medicine, Department of Radiology, Faculty of Medicine Siriraj Hospital, Mahidol University, Bangkok 10700, Thailand; potjanee.kan@mahidol.edu

**Keywords:** keratinocytes, melanocytes, paracrine factors, ultraviolet B, GCSF (granulocyte colony-stimulating factor), CCL20 (chemokine (C-C motif) ligand 20)

## Abstract

The skin microenvironment created by keratinocytes (KC) influences the stress responses of melanocytes (MC) to UVB insults. This study employed RNA sequencing analysis as well as in vitro and in vivo models to elucidate the underlying mechanisms. Our RNA-Seq analysis revealed a statistically significant upregulation of GCSF and CCL20 genes in UVB-irradiated KC, correlating with the protective effects of KC on MC responses to UVB exposure. Recombinant GCSF and CCL20 exhibited the most pronounced modulation of UVB-induced MC responses. These effects included the attenuation of apoptosis and reduction of ROS formation, along with the upregulation of tyrosinase and tyrosinase-related protein-1, which are involved in the melanogenic pathway. ELISA was also used to confirm that UVB could induce the secretion of GCSF and CCL20 from KC. A similar correlation between GCSF and CCL20 expression in KC and tyrosinase levels in MC was observed in UVB-irradiated mouse skin. Our study provides novel insights into the protective role of GCSF and CCL20 in the paracrine effects of KC on UVB-induced MC damage through the modulation of stress response pathways, the MITF-tyrosinase axis, and the regulation of p53. These findings have implications for the development of pharmacological strategies targeting KC-derived paracrine factors for the prevention of skin photodamage.

## 1. Introduction

Chronic exposure of the skin to UVB can disrupt melanocyte (MC) homeostasis and trigger stress responses, including apoptosis, DNA damage, and oxidative stress [1,2,3,4,5], ultimately contributing to the development of malignant melanoma [4,6]. Oxidative stress refers to the imbalance between the production of reactive oxygen species (ROS) and the skin’s ability to detoxify these reactive intermediates or repair the resulting damage [4]. UVB radiation is a significant source of oxidative stress as it increases ROS production in skin cells, leading to cellular damage, apoptosis, and inflammation, which contribute to photoaging and skin carcinogenesis [3,7]. Moreover, UVB radiation triggers an inflammatory response characterized by the release of pro-inflammatory cytokines, such as IL-1β, IL-6, and TNF-α from keratinocytes (KC) [3,8,9]. These cytokines mediate the recruitment of immune cells to the site of UVB exposure, leading to erythema, edema, and further ROS production, creating a cycle of inflammation and oxidative stress [2]. Chronic inflammation from repeated UVB exposure can lead to significant tissue damage and has been implicated in the development of skin cancers [1]. In addition, the microenvironment created by KC, which are epidermal cells, influences the homeostasis, function (including melanogenesis), and phenotype of the adjacent MC in response to ultraviolet radiation (UVR) via paracrine communication involving several soluble factors such as growth factors, cytokines, and hormones secreted from neighboring KC [7]. KC-derived paracrine factors influencing MC differentiation and inflammatory responses to UVB include endothelin-1 (ET-1), proopiomelanocortin (POMC)-derived peptides [such as α-melanocyte-stimulating hormone (α-MSH) and adrenocorticotropic hormone (ACTH)], and corticotrophin-releasing hormone (CRH) [3,10,11,12,13,14,15]. Exposure to UVR also promotes KC to secrete several pro-inflammatory cytokines, such as IL-1β, IL-6, and tumor necrosis factor (TNF)-α, which exert physiological and biological effects on MC [16]. Additionally, melanogenesis (pigmentation) serves as a vital adaptive protective response against further DNA damage induced by UVR [17]. Upon UVB exposure, KC-derived paracrine factors (e.g., α-MSH) can regulate melanogenesis and affect the survival and differentiation of MC via the microphthalmia-associated transcription factor (MITF) signaling pathway [18].

In contrast to previous studies focusing on differentiation and inflammation, our research aims to identify and characterize KC-derived paracrine factors that specifically modulate MC responses to UVB-induced cellular stress. Since the crosstalk between the KC microenvironment and MC plays a critical role in regulating skin homeostasis, identifying key KC-derived paracrine factors could lead to the development of biomarkers for predicting UVR sensitivity and photodamage risk. This knowledge could also pave the way for novel and effective strategies for skin cancer prevention.

In this study, we aimed to identify KC-derived paracrine factors that protect against UVB-induced MC damage. By examining the secreted factors from different epidermal and dermal cell sources that showed correlated UVB-protective effects, we identify candidate paracrine factors from transcriptomic profiling. We then confirmed the identity of paracrine factors that exhibit the strongest protective effect against UVB in MC using recombinant proteins. Finally, the proposed paracrine interactions between KC and MC were validated in vivo using a mouse skin model.

## 2. Materials and Methods

### 2.1. Cell Culture

Primary human epidermal melanocytes (MC) and primary human epidermal keratinocytes (KC) were obtained from Invitrogen (Waltham, MA, USA). MC were cultured in medium 254 (#M-254-500) supplemented with human melanocyte growth supplement (HMGS) according to the manufacturer’s instructions. KC were cultured in medium 154 (#M-154CF-500) supplemented with human keratinocyte growth supplement (HKGS). Human keratinocyte (HaCaT) cells (Cell Lines Service, Heidelberg, Germany) were grown in high-glucose (4.5 g/L) Dulbecco’s modified Eagle’s medium (DMEM) and Ham’s F-12 (DMEM/F-12) supplemented with 10% fetal bovine serum and 1% penicillin (100 U/mL)/streptomycin (100 mg/mL). Primary human dermal fibroblasts (HDF) were grown in high-glucose (4.5 g/L) DMEM supplemented with 10% fetal bovine serum, 1% 200 mM Glutamax, and 1% antibiotic-antimycotic solution (Mediatech, Inc., Manassas, VA, USA). Primary mouse embryonic fibroblast cells (NIH3T3) and epidermoid carcinoma cells (A431) were cultured in DMEM supplemented with 10% fetal bovine serum and 1% antibiotic-antimycotic solution (Mediatech, Inc., Manassas, VA, USA). All cells were maintained at 37 °C in a humidity of 5% CO_2_ (PCO_2_ = 40 Torr) using a Forma Scientific CO_2_ Water-Jacketed Incubator.

### 2.2. Preparation of Cell-Derived Conditioned Medium (CM)

KC, HaCaT, HDF, NIH3T3, and A431 were seeded at a density of 5 × 10^5^ cells/well in 6 cm^2^ dishes for general cell culture and expansion. CM was prepared at a cell density of 21 × 10^3^ cells/cm^2^ for all experiments to determine the effects of CM on apoptosis, ROS formation, and melanogenesis in MC. HDF, NIH3T3, and A431 supernatants were used as non-keratinocyte groups to compare the paracrine effects between keratinocyte and non-keratinocyte cells. Cells were irradiated with UVB (125 mJ/cm^2^) under a thin layer of Dulbecco’s Phosphate-Buffered Saline (DPBS) and then replaced with DMEM, that where then collected at 12 h post-irradiation and used as KC-CM, HaCaT-CM, HDF-CM, NIH3T3-CM, and A431-CM for treatment of MC. MC were pre-incubated with these CM samples for 2 h before UVB irradiation. After irradiation, the same culture medium that had been removed before irradiation was used to feed the irradiated cells again. Cells were harvested at 1 h following 62.5 mJ/cm^2^ UVB irradiation for determination of ROS formation, 12 h after 250 mJ/cm^2^ irradiation for caspase-3 activation, and 12 h after 125 mJ/cm^2^ irradiation for melanin content and tyrosinase activity.

### 2.3. UVB Irradiation

MC, KC, HaCaT, HDF, NIH3T3, and A431 cells were irradiated with UVB under a thin layer of DPBS. UV intensity, determined at a distance of 21 cm from the UVR lamp, was 1 W/cm^2^ using a UV meter (Dr. Honle, Martinsried, Germany). Following our previous observations, cells were exposed for 22.5, 45 s, or 1 min 30 s to achieve a single dose of 62.5, 125, or 250 mJ/cm^2^, respectively [3]. Immediately after UVB exposure, DPBS was replaced by medium 254 without HMGS for MC and DMEM without FBS for KC, HaCaT, HDF, NIH3T3, and A431.

### 2.4. Recombinant Paracrine Factor Treatment

MC were seeded in 6-well plates (2 × 10^5^ cells/well). Fourteen candidate paracrine factors, including CSF3/GCSF (300-23-2 ug; ProSpec, Rehovot, Israel) CCL20 (300-29A-5 ug; ProSpec, Rehovot, Israel), ET-1 (ab158332; Abcam, Cambridge, MA, USA), PIGF (100-06-5 ug; ProSpec, Rehovot, Israel), CXCL8 (200-08M-5 ug; ProSpec, Rehovot, Israel), TNFRSF10B (CYT-069; ProSpec, Rehovot, Israel), CXCL2 (300-39-2 ug; ProSpec, Rehovot, Israel), IL-6 (200-06-5 ug; ProSpec, Rehovot, Israel), IL-16 (200-16A-2 ug; ProSpec, Rehovot, Israel), BMP2 (120-02-2 ug; ProSpec, Rehovot, Israel), PSPN (450-12-5 ug; ProSpec, Rehovot, Israel), IL36G (CYT-160; ProSpec, Rehovot, Israel), EREG (100-04-5 ug; ProSpec, Rehovot, Israel), DKK1 (120-30-2 ug; ProSpec, Rehovot, Israel) were diluted in water. MC were incubated with the candidate paracrine factors at different concentrations (0.3, 1, 3, and 9 nM) for 2 h before UVB irradiation. The dissociation constant (*K*_D_) of the recombinant paracrine factors was used to determine the concentrations for assessing active caspase-3, oxidant formation, and melanin content in UVB-irradiated MC.

### 2.5. Determination of Intracellular Oxidant Formation by Flow Cytometry

2′,7′-Dichlorodihydrofluorescein diacetate (DCFH-DA) was used to determine intracellular reactive oxygen species (ROS). The principle of the DCFH-DA method is to detect the intensity of green fluorescence that occurs after deacetylation and the subsequent oxidation product of the probe. DCFH-DA penetrates cells and accumulates mainly in the cytosol, where it is hydrolyzed to DCFH, which is further oxidized by oxidants (e.g., H_2_O_2_) to fluorescent 2,7-dichlorofluorescein (DCF). The fluorescence intensity reflected overall oxidative stress and oxidant formation in the cells and was visualized and detected by flow cytometric analysis. At 1 h post-irradiation, MC were washed and incubated in DPBS containing 20 μM H_2_DCFDA at 37 °C for 30 min. The cells were then analyzed by flow cytometry using a fluorescence-activated cell sorter (BD FACS Calibur^TM^) (BD Biosciences, Franklin Lakes, NJ, USA) as previously described [19].

### 2.6. Measurement of Active Caspase-3

Caspase-3 is a key mediator of programmed cell death (apoptosis). In apoptotic cells, caspase-3 can be activated by both extrinsic (death ligand) and intrinsic (mitochondrial) pathways. In cells undergoing apoptosis, caspase-3 is an essential protease that is activated in the early stages of apoptosis and is synthesized as an inactive pro-enzyme.

Active caspase-3 was measured using the PE Active Caspase-3 Apoptosis Kit (BD Biosciences, Franklin Lake, NJ, USA) according to the manufacturer’s instructions. At 12 h post-irradiation, MC were washed, fixed, and permeabilized with Cytofix/CytopermTM for 30 min. Cells were washed with Perm/Wash TM Buffer, stained in the dark with the rabbit anti-active caspase-3 antibody (clone C92-605) (BD Biosciences, Franklin Lake, NJ, USA), and analyzed by flow cytometry as described in our previous report [3].

### 2.7. Determination of Melanin Content and Tyrosinase Activity

The melanin content and tyrosinase activity were determined in MC at 12 h post-irradiation. Melanin and tyrosinase activities monitored by dopachrome formation were measured spectrophotometrically at 475 using a spectrophotometer. The amount of melanin (μg/mg protein) was calculated by comparison with a standard curve generated with synthetic melanin. Tyrosinase activity (unit/mg protein) was calculated by comparison with a standard curve using tyrosinase (2034 U/mg) as previously described [20].

### 2.8. Quantitative Real-Time Reverse Transcriptase-Polymerase Chain Reaction (RT-PCR) for Determination of mRNA Expression

The mRNA levels of CRH, CRHR1, ET-1, POMC, tyrosinase, and TRP1 were determined by RT-PCR. Cells were harvested at 6 h post-irradiation, and total RNA was isolated using the Illustra RNAspin Mini RNA Isolation Kit (GE Healthcare, Amersham, UK). Reverse transcription was carried out using the Improm-II Reverse Transcriptase (Promega, Medison, WI, USA) under the conditions described in the kit manual. The primers for PCR were designed using the Primer Express software version 3.0 (Applied Biosystems, Foster City, CA, USA). The mRNA level was calculated by normalization against the expression level of GAPDH mRNA. The mean Ct value of mRNA expression in cDNA of each sample was compared with the mean Ct value of GAPDH determinations from the same cDNA samples, as described in our previous report [3]. The sequences of the PCR primers used are provided in Supplementary Table S1.

### 2.9. RNA Sequencing Analysis (RNA-Seq)

RNA was extracted using Trizol, purified by Ambion RNA isolation kit (Invitrogen), and treated with PureLink DNase set (Invitrogen). The RNA samples were quantified by measuring absorbance at 260 nm using the Qubit RNA HS assay kit. The integrity of RNA was analyzed by running on agarose gel electrophoresis and measuring RIN value with an Agilent 2100 Bioanalyzer (Agilent Technologies, Palo Alto, CA, USA). All samples showed high RNA integrity with intact 28sRNA, intact 16sRNa, and RNA integrity number (RIN) >8.

Total RNA of each sample was quantified and qualified by an Agilent 2100 Bioanalyzer (Agilent Technologies, Palo Alto, CA, USA), NanoDrop (Thermo Fisher Scientific Inc., Waltham, MA, USA), and 1% agarose gel. One μg total RNA with the RIN value above seven was used for the following library preparation.

Next-generation sequencing library preparations were constructed according to the manufacturer’s protocol (NEBNext^®^ Ultra™ RNA Library Prep Kit for Illumina^®^) (New England Biolabs, Ipswich, MA, USA). The poly (A) mRNA isolation was performed using the NEBNext Poly (A) mRNA Magnetic Isolation Module (NEB). The mRNA fragmentation and priming were performed using NEBNext First Strand Synthesis Reaction Buffer and NEBNext Random Primers. First-strand cDNA was synthesized using ProtoScript II Reverse Transcriptase, and the second-strand cDNA was synthesized using the Second Strand Synthesis Enzyme Mix. The purified (by AxyPrep Mag PCR Clean-up (Axygen, Union City, CA, USA)) double-stranded cDNA was then treated with End Prep Enzyme Mix to repair both ends and add a dA-tailing in one reaction, followed by a T-A ligation to add adaptors to both ends. Size selection of adaptor-ligated DNA was then performed using AxyPrep Mag PCR Clean-up (Axygen), and fragments of ~360 bp (with the approximate insert size of 300 bp) were recovered. Each sample was then amplified by PCR for 11 cycles using P5 and P7 primers, with both primers carrying sequences that can anneal with flow cells to perform bridge PCR, and the P7 primer has a six-base index allowing for multiplexing. The PCR products were cleaned up using AxyPrep Mag PCR Clean-up (Axygen, Union City, CA, USA), validated using an Agilent 2100 Bioanalyzer (Agilent Technologies, Palo Alto, CA, USA), and quantified using the Qubit 2.0 Fluorometer (Invitrogen, Carlsbad, CA, USA).

Then libraries with different indices were multiplexed and loaded on an Illumina HiSeq instrument according to the manufacturer’s instructions (Illumina, San Diego, CA, USA). Sequencing was carried out using a 2 × 150 bp paired-end (PE) configuration; image analysis and base calling were conducted by the HiSeq Control Software (HCS) (v2.2.38) + OLB + GAPipeline-1.6 (Illumina) on the HiSeq instrument.

Sequencing was performed on the Illumina HiSeq platform in a 2 × 150 bp paired end (PE) configuration, with 2.0 Gb of raw data per sample. Base-calling is performed by Illumina RTA software (v1.13) in a sequencer, and further demultiplexing is performed by Illumina bcl2fastq 2.17 software based on the index information. The number of reads and quality score (Q30) are also counted.

Alignment of reads to the human reference (hg19) was completed using STAR version v2.5.3a [21] on the Basepair website (https://www.basepairtech.com/), accessed on 13 July 2019. Other pipelines were used, including ‘sambamba’ (v0.6.6) [22] for sorting the BAM files, ‘samtools’ (v1.6) [23] for indexing the BAM files, ‘fastp’ (v0.19.4) [24] for the read trimming and QC, and ‘subread’ (v1.6.2) [25] for the gene/isoform quantification. Trimming of low-quality base pairs was completed using the Phred quality score (Q score) at ten as the cut-off [26]. Differential gene expression was completed using the BioJupies platform (https://biojupies.cloud), accessed on 16 July 2019. [27]. A statistically significant level was considered if the adjusted *p*-value was <0.05.

### 2.10. Determination of GCSF and CCL20 Levels by ELISA

GCSF and CCL20 levels in culture supernatants were determined using competitive enzyme immunoassay kits (DCS50 and DM3A00, R&D Systems, Inc., Minneapolis, MN, USA) according to the manufacturer’s instructions. Samples of GCSF and CCL20 standards were added to the immunoplate pre-coated with secondary antibodies. Anti-GCSF and anti-CCL20 antibodies were added, followed by a biotinylated peptide. The biotinylated peptide then interacted with streptavidin-horseradish peroxidase (HRP), which catalyzed the substrate solution.

### 2.11. Determination of UVB-Induced GCSF, CCL20, Tyrosinase, p-MITF, and p-p53 Protein Expressions In Vivo

All animal experiments were reviewed and approved by the Siriraj Animal Care and Use Committee (SiACUC), COA No.: 004/2563. Three-week-old female BALB/c wild-type mice were purchased from the National Laboratory Animal Center, Mahidol University and maintained under controlled conditions (25 ± 2 °C with 55 ± 5% relative humidity on a 12-h light:12-h dark cycle) using an isolator caging system. Water and food diet were available ad libitum during the experimental period, as previously described. Mice were randomized into four groups of 3 mice. Group I (control), without UVB exposure. Group II and III, UVB at 250 mJ/cm^2^/session 2 and 3 times a week (a cumulative total dose of 500 and 750 mJ/cm^2^, respectively). Mice in both the experimental and control groups were shaved before UVB irradiation to ensure uniform UVB exposure to the skin and to avoid interference from hair. Mice were anesthetized by intraperitoneal (i.p.) injection of 100:10 mg/kg of ketamine/xylazine cocktail. Dorsal skin was removed at 12 h after the last exposure of UVB, embedded in Tissue-Tek^®^ OCT compound and directly snap-frozen (liquid N_2_), and stored at −80 °C until microtome sectioning. Skin thickness was assessed by hematoxylin and eosin (H and E). CCL20, GCSF, tyrosinase, phosphorylated MITF (p-MITF), phosphorylated p53 (p-p53), pan-cytokeratin (keratinocyte marker), and gp100 (melanocyte marker) were assessed by immunofluorescence (IF) staining. Image analysis was performed using Image J as previously described [19,28,29].

### 2.12. Hematoxylin-Eosin (H and E) Staining and Analysis of Skin Thickness

Frozen tissues were collected at 12 h after UVB irradiation and sectioned using Cryostat (Thermo scientific, Waltham, MA, USA) for 8 μm per 1 section and then were fixed in ice-cold acetone and air-dried for 30 min at room temperature. H and E staining was performed for the histological evaluation of epidermal thickness. Tissue sections were washed in distilled water for 2 min, incubated with hematoxylin for 4 min, and then washed in distilled water for 10 min.

The slides were then incubated with eosin for 1 min and 95% alcohol for 1 min. The slides were dehydrated with 95% alcohol (15 s), 2 changes of absolute alcohol (15 s each), 2 changes of acetone (15 s each), and 3 changes of xylene (15 s). Stained slides were mounted in permanent aqueous mounting medium with a coverslip for the analysis under the bright-field microscopy as previously described [28].

### 2.13. Immunofluorescence Analysis of CCL20, GCSF, and Tyrosinase, p-MITF, and p-p53

Tissue sections were blocked with 2% bovine serum albumin (BSA) in phosphate-buffered saline (PBS) for 30 min. After blocking, the slides were incubated with primary antibodies against CCL20, GCSF, tyrosinase, p-MITF, p-p53, pan-cytokeratin, and gp100, followed by incubation with Alexa Fluor^®^-conjugated secondary antibodies and DAPI for nuclear counterstaining. The details of the antibodies used, including dilutions and catalog numbers, are provided in Supplementary Table S1.

### 2.14. Statistical Analysis

Data are expressed as mean ± standard deviation of the mean (SD) of at least three separate experiments (*n* ≥ 3) performed on different days. Statistical significance of differences between different groups was evaluated by an independent *t*-test (student’s; 2 populations) or one-way analysis of variance (ANOVA) followed by Dunnett’s tests, where appropriate, using Prism(v9) (GraphPad Software Inc., San Diego, CA, USA).

## 3. Results

### 3.1. Paracrine Modulation of UVB-Induced Stress Responses in MC

**Figure 1:**
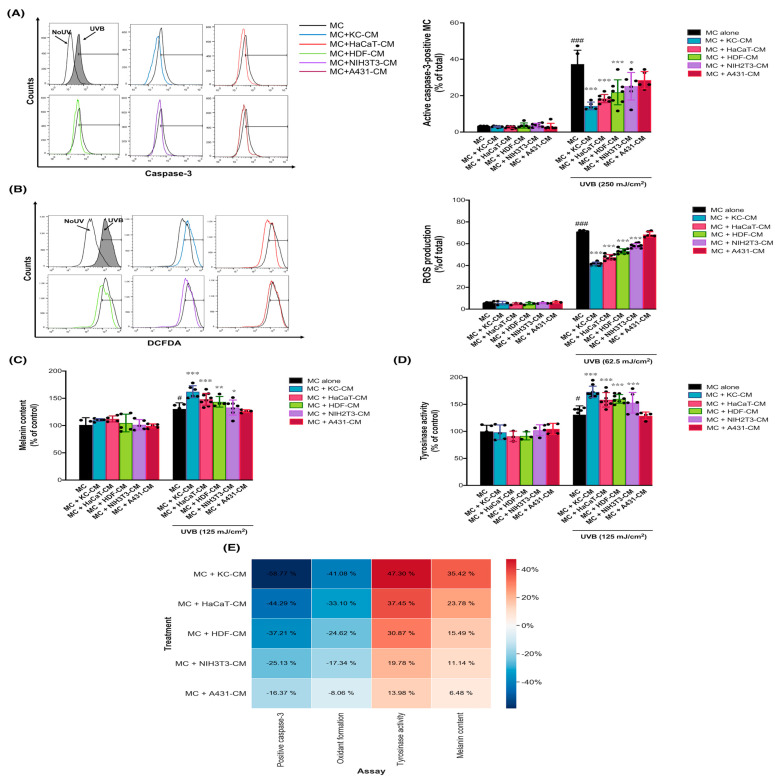
The paracrine effects of the different epidermal and dermal cell sources on UVB-induced stress responses in MC. The effects of UVB on caspase3 activation (**A**), ROS formation (**B**), melanin content (**C**), and tysosinase activity (**D**) in MC pretreated with CM from KC, HaCaT, HDF, NIHT3T, and A431 irradiated with UVB (125 mJ/cm^2^) for 2 h. The statistical significance of differences between UVB-irradiated MC and UVB-irradiated MC + KC − CM, MC + HaCaT − CM, MC + HDF − CM, MC + NIH3T3 − CM, and MC + A431 − CM was evaluated by one-way ANOVA followed by Dunnett’s test (* *p* < 0.05; ** *p* < 0.01; *** *p* < 0.001 versus UVB-irradiated MC; # *p* < 0.05; ### *p* < 0.001 versus unirradiated MC). A heat map representing color-coded expression levels of the paracrine protective effects of KC, HaCaT, HDF, NIHT3T, and A431 cells on UVB-induced MC responses is shown (**E**). The red color indicated the inhibitory action of the paracrine protective effects, while the gray color indicated the activating action of the paracrine protective effects.

To investigate the paracrine modulation of UVB-induced stress responses in MC, we included five epidermal and dermal cell sources: KC, HaCaT, HDF, NIH3T3, and A431 cells in our study. These were used as a comparative model to evaluate the effects of the microenvironment, which was predominantly created by KC, on the responses of MC to UVB-induced stress. Our aim was to identify which epidermal and dermal cell sources, through their derived paracrine factors, predominantly influenced the phenotypic changes in MC following UVB irradiation. Initially, we induced paracrine release by subjecting the epidermal and dermal cell sources to UVB irradiation (125 mJ/cm^2^) and collected the cells 12 h later. Subsequently, MC were exposed to the conditioned media (CM) from these epidermal and dermal cell sources for 2 h prior to UVB irradiation. In the absence of CM, UVB irradiation led to a statistically significant increase in caspase-3 activation (37 ± 6% of positively stained cells, or a 12-fold increase relative to the no-UV control). This corresponds to a median survival rate of 65% following UVB exposure at 125 mJ/cm^2^, indicating a substantial reduction in cell viability due to UVB-induced apoptosis (Figure 1A). We first demonstrated that, at 1 h post-irradiation, UVB irradiation significantly induced active caspase-3 (Figure 1A) and oxidative stress, as evidenced by ROS formation (71 ± 1% of positively stained cells or a 12-fold increase relative to the no-UV control) in MC (Figure 1B). Moreover, UVB exposure resulted in a substantial rise in melanin content (125 ± 18 µg/mg protein or a 1.25-fold increase relative to the no-UV control) (Figure 1C) and tyrosinase activity (120 ± 25 units/mg protein or a 1.2-fold increase relative to the no-UV control) (Figure 1D) in MC. Following UVB irradiation, MC pretreated with CM from various sources exhibited a marked reduction in UVB-induced active caspase-3. Specifically, KC (14 ± 2% or a 2.6-fold decrease relative to the UVB control), HaCaT (18 ± 2% or a 2-fold decrease relative to the UVB control), HDF (22 ± 5% or a 1.7-fold decrease relative to the UVB control), and NIH3T3 (25 ± 3% or a 1.5-fold decrease relative to the UVB control). Moreover, post-UVB exposure, MC pre-incubated with CM from different cell sources led to a decrease in ROS production. Specifically, KC (41 ± 1% or a 1.7-fold decrease relative to the UVB control), HaCaT (49 ± 2% or a 1.5-fold decrease relative to the UVB control), HDF (53 ± 2% or a 1.3-fold decrease relative to the UVB control), and NIH3T3 (59 ± 2% or a 1.3-fold decrease relative to the UVB control). CM-treated MC showed significantly lower levels compared to untreated MC (Figure 1A,B). Furthermore, post-UVB exposure, MC pre-incubated with CM from different cell sources exhibited increased melanin content and tyrosinase activity. Notably, KC (172 ± 11 µg/mg protein or a 1.7-fold increase relative to the UVB control), HaCaT (155 ± 11 µg/mg protein or a 1.5-fold increase relative to the UVB control), HDF (140 ± 12 µg/mg protein or a 1.4-fold increase relative to the UVB control), and NIH3T3 (136 ± 14 µg/mg protein or a 1.4-fold increase relative to the UVB control). Additionally, MC pre-incubated with CM from different cell sources showed an increase in tyrosinase activity after UVB exposure. Specifically, KC (178 ± 13 units/mg protein or a 1.7-fold increase relative to the UVB control), HaCaT (160 ± 13 units/mg protein or a 1.6-fold increase relative to the UVB control), HDF (158 ± 8 units/mg protein or a 1.6-fold increase relative to the UVB control), and NIH3T3 (145 ± 24 units/mg protein or a 1.4-fold increase relative to the UVB control) (Figure 1C,D).

To further validate our findings, we performed additional experiments to confirm the effects of UVB exposure (31.25, 62.5, and 125 mJ/cm^2^) on melanin content in MC and KC. The results showed that UVB exposure significantly increased melanin content in MC in a dose-dependent manner. However, UVB exposure did not significantly induce melanin content in KC (Figure S1).

Across various epidermal and dermal cell types, CM derived from skin cells including KC, HaCaT, HDF, and NIH3T3 (except for A431 cells) significantly suppressed caspase-3 activation and ROS formation while enhancing tyrosinase activity and melanin levels in UVB-irradiated MC (Figure 1E). Importantly, KC exhibited the most potent paracrine modulatory effects on UVB-induced stress responses, including photodamage and melanogenesis. To further validate our results, we assessed the paracrine modulatory effects of KC-derived CM at different cell densities. We observed that different cell plating densities, such as 1:5 (MC:KC-CM) and 1:10 (MC:KC-CM), provided statistically significant paracrine protective effects against UVB-induced apoptosis in MC. This finding highlights the cell number-dependent nature of the protective effect of KC-derived CM against MC damage. Consequently, we selected the MC:KC-CM ratio of 1:5 for subsequent experiments, as this condition exhibited the most robust modulatory effects against stress responses in UVB-irradiated MC (Figure S2).

### 3.2. Identification of Key Paracrine Factors in KC Modulating UVB-Mediated Mc Responses

To pinpoint the key paracrine factors in KC responsible for their modulatory effects on UVB-induced responses in MC, we conducted a systematic analysis. We began by profiling the transcriptomes of all four dermal cell types post-UVB irradiation to explore potential candidate paracrine factors. To ensure the accuracy of gene expression timing, we initially assessed the expressions of CRH, CRHR1, ET-1, and POMC genes in KC using RT-PCR at various time points (2, 4, 6, 8, and 12 h) post-UVB irradiation (125 mJ/cm^2^) (Figure S3A). These genes, which encode secreted factors from KC, exhibited maximal expression changes at 6 h post-irradiation. Subsequently, we compared the mRNA expression of these paracrine factors across different dermal cell sources, including KC, HaCaT, HDF, and A431 cells. KC displayed the most statistically significant increase in CRHR1, ET-1, and POMC, while HaCaT only showed an increase in ET-1. Conversely, HDF and A431 exhibited negligible changes in these paracrine factors (Figure S3B).

**Figure 2:**
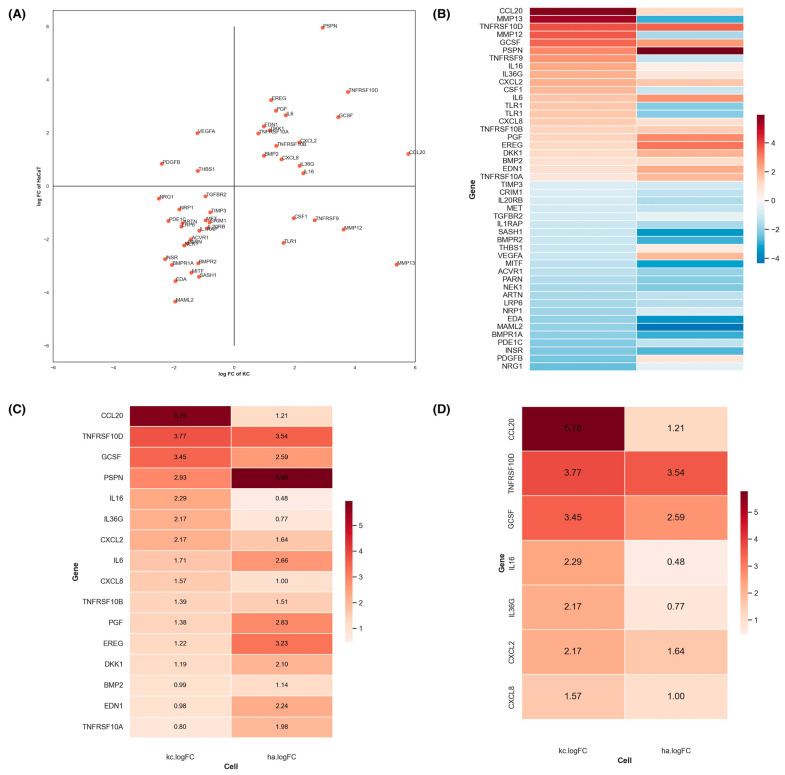
The effects of UVB on gene expression of secreted paracrine factors in KC and HaCaT cells. The relative differentially expressed genes of UVB-irradiated KC and HaCaT (ha) cells were calculated (**A**). The data are presented as a log2 fold change of UVB-irradiated cells compared to unirradiated cells (control cells). The *x*-axis shows the differentially expressed genes of UVB-irradiated KC, whereas the *y*-axis displays the differentially expressed genes of UVB-irradiated HaCaT cells. A heatmap analysis of differentially expressed genes in UVB-irradiated KC and HaCaT (ha) cells is shown (**B**). The data are presented as a log2 fold change of UVB-irradiated cells compared to unirradiated cells (control cells). The red color represents up-regulated genes, while the blue color represents non-differentially expressed genes. A total of 1730 genes were identified as differentially expressed (FDR ≤ 0.05) between UV-treated and control cells. UVB treatment of KC and HaCaT cells caused a statistically significant expression of 46 paracrine factor genes compared to unirradiated cells (**B**). UVB (125 mJ/cm^2^) treatment caused 16 up-regulated transcripts in both KC and HaCaT cells (**C**). Seven paracrine factor genes were markedly upregulated in UVB-treated KC compared to UVB-treated HaCaT cells (**D**).

To further narrow down our focus, we employed RNA-seq analysis to identify differential gene expression between UVB-irradiated and unirradiated KC and HaCaT cells at the 6 h time point. A total of 1730 genes were identified as differentially expressed (FDR ≤ 0.05) (Supplementary Table S3), encompassing both upregulated and downregulated genes. From this list, we specifically identified 46 genes encoding secreted proteins (Figure 2A,B), including inflammatory mediators and cytokines [30]. Among these, 16 genes were upregulated in both KC and HaCaT cells (Figure 2C). Notably, the top 7 paracrine factor genes most strongly upregulated in UVB-irradiated KC included chemokine (C-C motif) ligand 20 (CCL20), TNF Receptor Superfamily Member 10b (TNFRSF10D), granulocyte colony-stimulating factor (GCSF/CSF3), interleukin 6 (IL6), interleukin 36 gamma (IL36G), chemokine (C-X-C motif) ligand 2 (CXCL2), and chemokine (C-X-C motif) ligand 8 (CXCL8) (Figure 2D). It is important to note that HaCaT cells are defective in both TP53 alleles, which may explain the lack of complete overlap between keratinocytes and HaCaT cells, with only 16 of the 46 genes showing concordant expression [31,32]. These candidate genes became the focal point for further validation of their paracrine modulatory effects on UVB-mediated MC responses.

### 3.3. Role of Candidate Paracrine Factors in Modulating UVB-Induced Stress Responses in MC

To validate the role of our identified candidate paracrine factors in mitigating UVB-induced stress responses in MC, we conducted experiments utilizing recombinant paracrine factors. MC were treated with each of the 14 candidate paracrine factors [GCSF, CCL20, endothelin-1 (ET-1), placental growth factor (PIGF), CXCL8, TNFRSF10B, CXCL2, IL-6, interleukin 16 (IL-16), bone morphogenetic protein 2 (BMP2), persephin (PSPN), IL36G, epiregulin (EREG), the Dickkopf-related protein 1 (DKK)] prior to exposure to UVB irradiation. Our findings revealed that six of these factors, namely GCSF, CCL20, ET-1, PIGF, CXCL8, and TNFRSF10B, at concentrations ranging from 0.3 to 9 nM, effectively suppressed caspase-3 activation in UVB-irradiated MC (Figure S4A). Similarly, pre-treatment with GCSF, CCL20, ET-1, CXCL8, TNFRSF10B, or BMP2 (0.3–9 nM for all six ligands) led to a reduction in ROS formation in UVB-irradiated MC (Figure S4B). Furthermore, the addition of GCSF (1–9 nM), CCL20 (3–9 nM), ET-1 (1–9 nM), PIGF (0.3–9 nM), or CXCL8 (9 nM) stimulated melanin content in UVB-irradiated MC (Figure S4C).

To support the biological significance of these findings, we compared the magnitude of changes induced by GCSF and CCL20 with those of previously reported paracrine factors such as ET-1, CRH, and α-MSH. Previous studies have shown that ET-1 and α-MSH significantly modulate apoptosis and stress responses in UV-irradiated MC, with ET-1 reducing apoptosis and oxidative stress by approximately 1–4-fold. Moreover, α-MSH significantly inhibits apoptosis and stress responses in UV-irradiated MC by approximately 2–3-fold [12]. Additionally, α-MSH has been reported to reduce DNA damage and apoptosis in UVB-irradiated MC by 1–2-fold [10]. In our study, GCSF and CCL20 demonstrated comparable effects, with GCSF reducing UVB-induced caspase-3 activation by approximately 2-fold and ROS formation by 2.7-fold and CCL20 reducing caspase-3 activation by 1.8-fold and ROS formation by 2.3-fold. These results suggest that the paracrine effects of GCSF and CCL20 are biologically impactful and similar in magnitude to those of established paracrine factors such as ET-1 and α-MSH. Furthermore, CRH has been shown to modulate stress responses in MC, particularly under UV exposure, by reducing oxidative stress and apoptosis [13]. The comparable effects of GCSF and CCL20 in our study further validate their potential role in the protective paracrine network against UVB-induced stress in MC. These comparisons highlight the relevance and biological significance of GCSF and CCL20 as key paracrine factors in protecting MC against UVB-induced damage.

**Figure 3:**
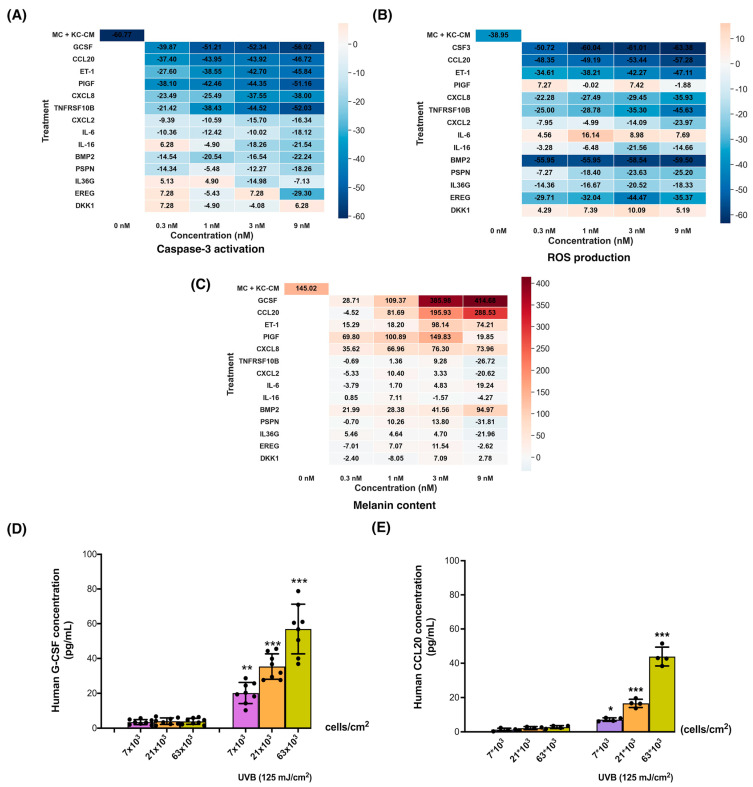
The protective effects of paracrine factors on UVB-induced apoptosis, ROS formation, and melanogenesis in MC. A heat map representing the color-coded expression levels of the paracrine protective effects of KC and 14 recombinant paracrine factors on UVB-induced apoptosis (**A**), ROS production (**B**), and melanin content (**C**) in MC cells was created. The blue color represented the inhibitory action of paracrine protective effects, while the red color represented the activating action. We examined the effects of UVB (125 mJ/cm^2^) on GCSF and CCL20 levels in KC cells. At 12 h post-irradiation, CM from KC at three cell concentrations (7 × 10^3^, 21 × 10^3^, 63 × 10^3^ cells/cm^2^) were collected, and the concentrations of GCSF (**D**) and CCL20 (**E**) were measured. The data are expressed as mean ± SD. The statistical significance of differences between UVB-irradiated KC at different cell concentrations (7 × 10^3^, 21 × 10^3^, 63 × 10^3^ cells/cm^2^) was evaluated by one-way ANOVA followed by Dunnett’s test (* *p* < 0.05; ** *p* < 0.01; *** *p* < 0.001 versus unirradiated control KC).

We summarized the modulation of UVB-induced MC responses by different recombinant proteins using heatmap analysis (Figure 3A–C). Our results indicated that GCSF, CCL20, and ET-1 exhibited the ability to suppress caspase-3 activation and ROS formation as well as promote melanin content in UVB-irradiated MC. It is important to note that melanin synthesis is a highly ROS-generating process [33]. Therefore, the observed reduction in ROS levels in MC treated with GCSF and CCL20 might be due to the activation of protective mechanisms that counteract the ROS generated during melanogenesis. This reduction in ROS levels, despite the increase in melanin synthesis, suggests that GCSF and CCL20 not only promote melanogenesis but also enhance the antioxidant defenses of MC. This dual action could be a critical aspect of their protective role against UVB-induced damage, as it helps to mitigate oxidative stress while supporting the skin’s natural pigmentation response to UV exposure. To further corroborate our findings, we utilized ELISA to confirm that UVB irradiation induced the secretion of GCSF and CCL20 from KC. At 12 h post-irradiation, KC exposed to UVB (125 mJ/cm^2^) at various cell plating densities (7 × 10^3^, 21 × 10^3^, 63 × 10^3^ cells/cm^2^) exhibited a corresponding increase in GCSF (20 ± 6, 37 ± 6, 58 ± 10 pM) and CCL20 levels (7 ± 1, 17 ± 2, 44 ± 5 pM) (Figure 3D,E).

### 3.4. Modulatory Effects of Candidate Paracrine Factors on UVB-Induced Melanogenesis-Related Genes in MC

**Figure 4:**
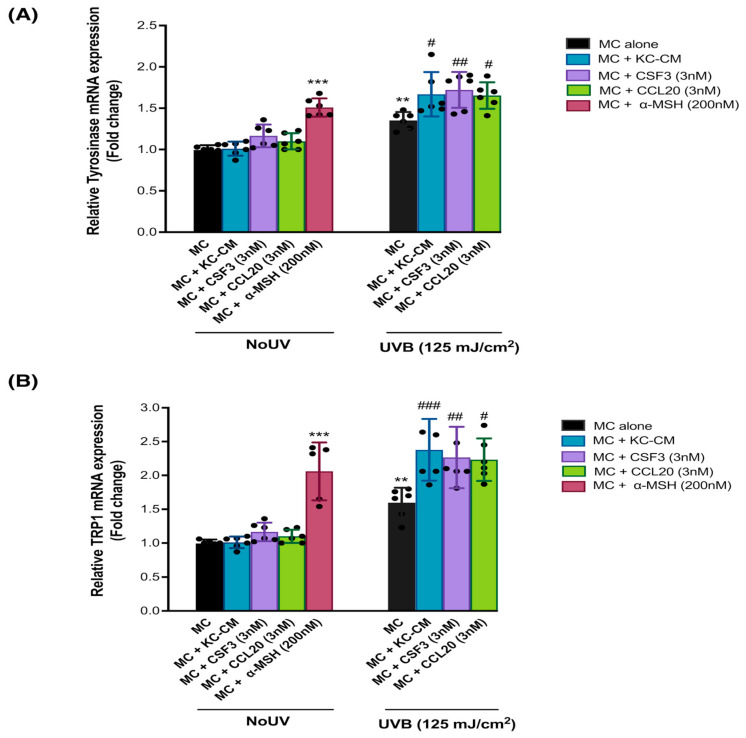
The protective effects of the paracrine factors on UVB-induced transcriptional activation of MITF in MC. We examined the effects of UVB (125 mJ/cm^2^) on tyrosinase (**A**) and TRP1 (**B**) mRNA expression in MC pretreated with CM from KC, irradiated with UVB (125 mJ/cm^2^), GCSF, CCL20 (9 nM), and α-MSH (200 nM). MC were harvested at 1 h after UVB irradiation for the determination of ROS formation. Data are expressed as mean ± SD. The statistical significance of differences was evaluated by one-way ANOVA followed by Dunnett’s test. ** *p* < 0.01; *** *p* < 0.001 versus unirradiated cells. The statistical significance of differences between UVB-irradiated MC and UVB-irradiated MC+KC-CM, as well as GCSF and CCL20, was evaluated by one-way ANOVA followed by Dunnett’s test (# *p* < 0.05; ## *p* < 0.01; ### *p* < 0.001 versus UVB-irradiated MC).

As previous results indicated the potent impact of paracrine factor ligands, particularly GCSF and CCL20, on MC in response to UVB-induced melanogenesis, we further investigated their effects on crucial melanogenesis-related genes, specifically tyrosinase and TRP-1 [17]. Our findings demonstrated that pretreatment with recombinant human GCSF and CCL20 (3 nM) led to a statistically significant upregulation of tyrosinase (Figure 4A) and TRP1 mRNA expression (Figure 4B) in UVB-irradiated MC. Notably, this upregulation of tyrosinase and TRP-1 mRNA was also observed in MC treated with KC-conditioned media (KC-CM) and in the positive control, α-MSH (200 nM). α-MSH is a well-known effective activator of melanogenesis-related genes, including tyrosinase and TRP-1.

### 3.5. Protective Effects of KC’s Paracrine Factors, GCSF, and CCL20, on UVB-Induced Skin Damage in Mice

**Figure 5:**
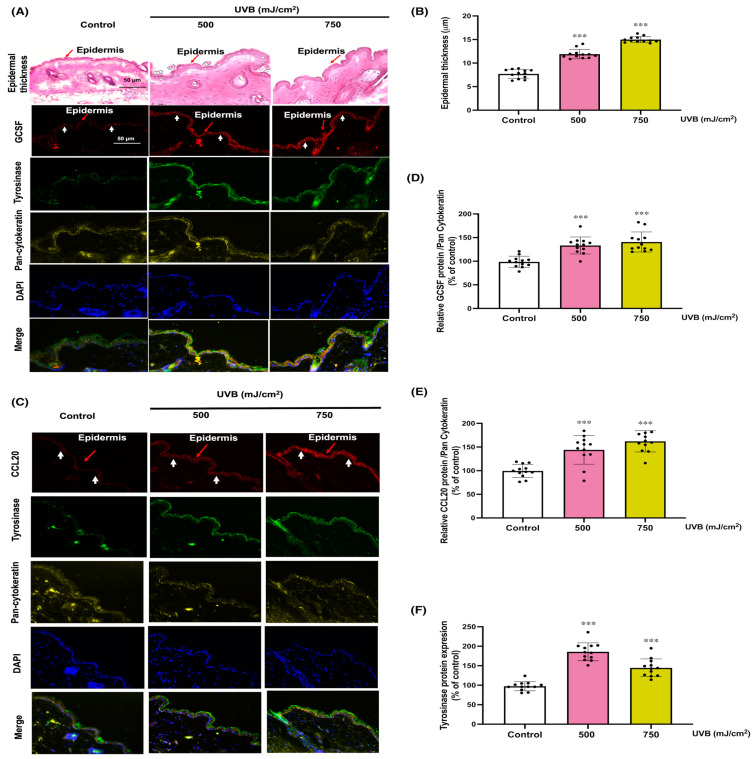
Epidermal thickness and the expression of GCSF, CCL20, and tyrosinase protein in UVB-treated mouse skin. Epidermal thickness (**A**), immunofluorescence of GCSF (**A**), CCL20 (**C**), tyrosinase (**A**,**C**), and pan-cytokeratin, a marker that identifies keratinocytes in all layers of the epidermis (**A**,**C**), were assessed 12 h following the final UVB exposure. Red arrows indicate the epidermal layer, while white arrows mark the dermal-epidermal junction, delineating the boundary between the epidermis and dermis. The summary graph shows the statistical analysis of epidermal thickness (**B**), and the relative protein levels of GCSF (**D**), and CCL20 (**E**), to pan-cytokeratin and tyrosinase (**F**). The data were quantified using ImageJ and GraphPad Prism software (v9) and are expressed as mean ± SD, with 1 N (dot) representing one area from four areas in a mouse. A mouse provides three N. The statistical significance of differences was evaluated by one-way ANOVA followed by Dunnett’s test. *** *p* < 0.001 versus non-irradiated group.

To evaluate the protective role of KC-derived paracrine factors, specifically GCSF and CCL20, in a physiological context, we employed mouse models of UVB-induced skin photodamage. We recognized that the transcriptional profiling of KC in skin can be influenced by surrounding non-KC cell types. Analysis of H and E-stained sections of mouse skin tissues revealed a substantial and dose-dependent increase in epidermal thickness (11.90 ± 1 µm and 14.99 ± 0.6 µm, representing a 1.5 and 1.9-fold increase, respectively) following UVB irradiation (500 and 750 mJ/cm^2^) (Figure 5A,B). These UVB doses were selected based on previous studies showing that higher doses effectively induce oxidative stress, inflammation, and skin damage [34], allowing us to evaluate the protective effect of GCSF and CCL20 on severe UVB-induced damage. The increase in epidermal thickness observed following UVB exposure in mouse skin is a well-documented adaptive response that enhances the skin’s barrier function and provides protection against further UV-induced skin damage. In addition to epidermal thickening, we also observed swelling of the dermis in response to UVB irradiation, which indicates underlying inflammatory processes. Furthermore, UVB exposure (750 mJ/cm^2^) induced the expression of GCSF and CCL20 in KC (Figure S6). Immunofluorescent staining showed a correlated increase in the abundance of both GCSF and CCL20 proteins along with melanogenic responses to UVB irradiation in mouse skin (Figure 5A,C–E). Moreover, UVB irradiation (500, 750 mJ/cm^2^) led to a dose-dependent increase in the protein expression of GCSF (133 ± 11%, 140 ± 21% of control, respectively, or a 1.3- and 1.4-fold increase relative to control), CCL20 (144 ± 30%, 162 ± 22% of control, respectively, or 1.4- and 1.6-fold increase relative to control), and tyrosinase (186 ± 23%, 145 ± 23% of control, respectively, or 1.8 and 1.4-fold increase relative to control) in KC and MC (Figure 5A,C–F). This observation indicated a positive correlation between the expressions of GCSF and CCL20 proteins in the epidermis and tyrosinase expression, the enzyme responsible for melanin synthesis in UVB-irradiated mouse skin.

**Figure 6:**
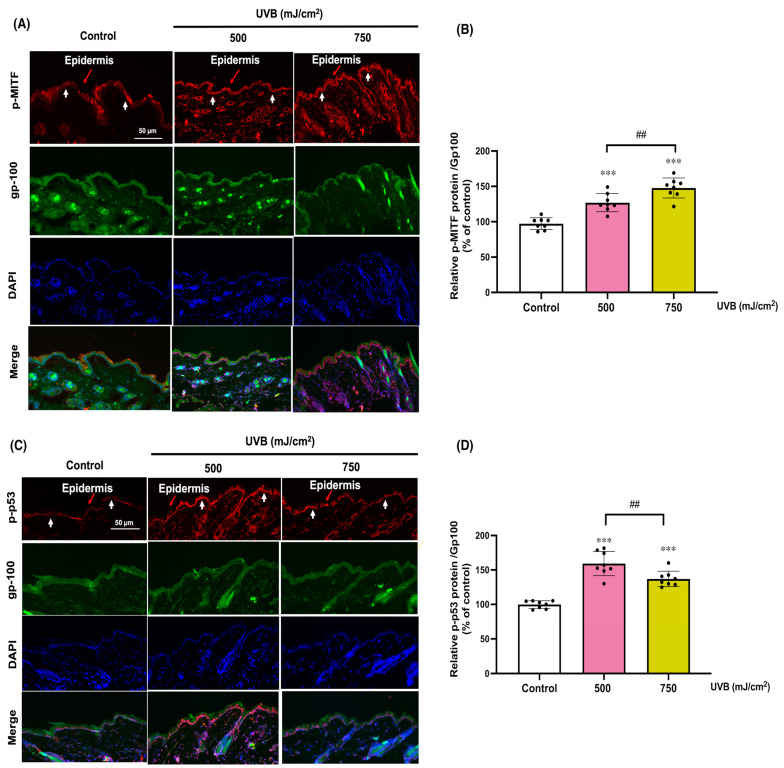
The expressions of phosphorylated MITF and p53 proteins in mouse skin exposed to UVB irradiation. The immunofluorescence of phosphorylated MITF (p-MITF) (**A**), phosphorylated p53 (p-p53) (**B**), and gp-100, a melanocyte marker, was assessed 12 h following the final UVB exposure. Red arrows indicate the epidermal layer, while white arrows mark the dermal-epidermal junction, delineating the boundary between the epidermis and dermis. The summary graph shows the statistical analysis of the relative protein levels of p-MITF (**C**), p-p53 (**D**), and gp-100. The data were quantified using ImageJ and GraphPad Prism software and are expressed as mean ± SD, with 1 N (dot) representing one area from four areas in a mouse. A mouse provides three N. *** *p* < 0.001 versus non-irradiated group by Student’s *t*-test. ## *p* < 0.01 versus UVB (500 mJ/cm^2^)-irradiated group by Student’s *t*-test.

We further assessed the activation of MITF, a key transcription factor regulating melanogenesis and MC stress responses, and p53, a master regulator of the cell stress response [35]. UVB irradiation (500 and 750 mJ/cm^2^) dose-dependently induced p-MITF (127 ± 13%, 148 ± 14% of control, respectively, or a 1.3 and 1.5-fold increase relative to control) and p-p53 (159 ± 18%, 137 ± 11% of control, respectively, or a 1.6 and 1.4-fold increase relative to control) (Figure 6A–D). These findings suggest that KC-derived paracrine factors, GCSF and CCL20, may play a regulatory role in protecting against UVB-induced photodamage by activating the melanogenic pathway, including MITF-tyrosinase signaling, and downregulating p53. Although both p-MITF and gp100 are involved in the melanogenic response to UVB, our results suggest that they do not colocalize due to their distinct cellular localizations, with p-MITF found in the nucleus and gp100 in cytoplasmic melanosomes, reflecting their sequential roles in melanogenesis.

## 4. Discussion

Our previous findings and existing research have underscored the protective role of KC against UVB-mediated photodamage in MC, involving the reduction of apoptosis, oxidative stress, DNA damage, and inflammatory responses, along with the promotion of melanogenesis [3,36]. In this study, we explored the crosstalk between MC and the KC-created microenvironment, aiming to identify key paracrine factors from KC that protect MC from UVB-induced photodamage.

By assessing various epidermal and dermal cell sources with differing protective effects against UVB-mediated photodamage in MC, we reasoned that the expression levels of these paracrine factors in KC should correspond to their protective effects. This approach led us to identify statistically significant paracrine factor genes, including CCL20, TNFRSF10D, GCSF, IL6, IL36G, CXCL2, and CXCL8. Moreover, our experiments with recombinant paracrine factors, including GCSF, CCL20, ET1, PIGF, and CXCL8, demonstrated their ability to dose-dependently suppress caspase-3 activation, reduce ROS formation, and stimulate melanogenesis in UVB-irradiated MC. The secretion of GCSF and CCL20 from KC-derived conditioned media after UVB exposure further confirmed their role in this protective mechanism.

MC, acting as stress sensors in the epidermis exposed to environmental threats, are subject to biological and physiological regulation by KC. This regulation serves as a photoprotective signature and represents a potential therapeutic target for UVB-induced photodamage. Soluble paracrine factors such as hepatocyte growth factor (HGF), Neuregulin-1 (NRG-1), transforming growth factor-β (TGF-β), and basic fibroblast growth factor (bFGF), produced by both KC and HDF, have previously been shown to regulate MC homeostasis [37,38]. Notably, commonly known UVB signature genes, such as ET-1 and POMC, have been suggested as key KC-derived paracrine factors that regulate MC homeostasis and function in response to UVR [11,39,40]. Our study identified a set of UVB-responsive genes whose upregulated transcripts, particularly at an early time point (6 h post-exposure), exceeded the expression levels of previously reported UVB signature genes. This indicates the dynamic nature of KC gene regulation in response to UVB exposure.

In addition, UVB radiation is known to trigger a robust inflammatory response in the skin, characterized by the release of various cytokines and chemokines from KC [3,8,9]. These inflammatory mediators play critical roles in the skin’s defense mechanism by recruiting immune cells to the site of damage and promoting repair processes [2]. Previous research has highlighted the roles of colony-stimulating factors such as CSF2 (GM-CSF) and GCSF (CSF3), along with CCL20, in regulating cutaneous homeostasis, tissue repair, hyperpigmentation, and inflammation [41,42,43,44]. GCSF has been associated with improved wound healing through anti-apoptotic and anti-inflammatory actions, while CCL20 has been implicated in the early stages of wound healing in UVB-induced epidermal injury models. Additionally, GM-CSF derived from KC has been linked to the control of mouse MC proliferation and differentiation in response to UVB-induced pigmented spots [45]. Our results revealed that GCSF and CCL20 could upregulate MITF target genes, including tyrosinase and TRP-1, involved in melanogenesis in UVB-irradiated MC. This study also demonstrated the upregulation of MITF activity and downregulation of p-53, a crucial transcription factor controlling various stress responses, including apoptosis, were associated with the increased expressions of GCSF and CCL20 in UVB-irradiated mouse skin. The levels of CCL20 and GCSF secreted in KC-CM increased in a UVB dose-dependent manner (Figure S5). Additionally, treatment with recombinant paracrine factors, CCL20 and GCSF (but not with other recombinant proteins), dose-dependently increased melanin content in MC. Consistent with the in vitro results, our in vivo study demonstrated that UVB exposure to mouse skin led to increased expressions of both CCL20 and GCSF in KC, which correlated with elevated tyrosinase protein levels in MC. Therefore, as observed in our study, among several UVB-responsive genes involved in paracrine signaling identified in KC, the paracrine factors GCSF and CCL20 might play a protective role in KC’s paracrine actions against UVB-induced stress responses, including oxidative stress, melanogenesis, and apoptosis, possibly through the regulation of MITF and p53 in MC. To further elucidate these mechanisms, genetic knockdown approaches should also be employed to investigate the specific role of MITF and p53 in mediating the protective effects of GCSF and CCL20. These would lead to a deeper understanding of how these paracrine factors modulate UVB-induced stress responses at the molecular level. Although the protective roles of GCSF and CCL20 in UVB-induced MC damage have been clarified, it is important to investigate whether these factors act synergistically or redundantly. It is possible that GCSF and CCL20 exhibit synergistic effects by simultaneously enhancing these protective mechanisms. Future studies will include simultaneous treatment with GCSF and CCL20 to evaluate possible additive effects on UVB-induced stress responses such as apoptosis, ROS formation, and melanogenesis. While our study sheds light on UVB-responsive genes in KC, further studies utilizing both in vitro and in vivo models with KC depleted of CCL20 and GCSF are required to further elucidate the specific mechanisms through which CCL20 and GCSF signaling regulate stress response in MC. Additionally, it is necessary to elucidate the intricate signaling pathways involved in the crosstalk between KC and MC. This could provide valuable insights into potential biomarkers for the development of targeted prevention and treatment strategies for UVB-induced skin conditions associated with MC damage.

High-throughput approaches have been instrumental in identifying UV-responsive genes and proteins in skin cells and mouse skin. However, it is crucial to acknowledge that paracrine factors, including growth factors and cytokines, can have dual roles, either promoting tissue repair or contributing to immune responses, inflammation, and carcinogenesis. Hence, additional investigations are warranted to assess clinical relevance and validate the specificity of UV signature genes in predicting skin photodamage and photocarcinogenesis risk.

## 5. Conclusions

In summary, our study highlights the significance of GCSF and CCL20 as major paracrine factors secreted by KC, contributing to KC’s paracrine protective effects on MC against UVB-induced stress responses by regulating the MITF-tyrosinase signaling pathway and p53. The UVB-responsive genes identified in KC hold promise as potential clinical UVB biomarkers for predicting skin susceptibility to photodamage. Furthermore, these UVB signature paracrine factors could be explored as pharmacological targets for developing KC-derived paracrine factors to prevent and treat skin photodamage.

## Data Availability

The authors confirm that the data supporting the findings of this study are available within the article and its Supplementary Materials.

## Supplementary Materials

The following supporting information can be downloaded at: https://www.mdpi.com/article/10.3390/cells13191661/s1. Figure S1: The effects of UVB on melanin content in MC and KC at 12 h after UVB irradiation; Figure S2: The effects of different cell ratios of KC, HaCaT, and HDF on UVB-induced caspase3 activation in MC at 12 h after UVB irradiation; Figure S3: The effects of UVB on genes encoding secreted paracrine factors (CRH, CRH1, ET-1, POMC) expression in KC, HaCaT, HDF, and A431 cells; Figure S4: The protective effects of paracrine factors on UVB-induced apoptosis, ROS formation, and melanogenesis in MC cells; Figure S5: The effects of UVB on GCSF and CCL20 levels in KC cells at 12 h after UVB irradiation; Figure S6: The expression of G-CSF and CCL20 protein in mouse skin exposed to UVB irradiation; Table S1: Primer sequences; Table S2: Primary and Secondary Antibodies Used for Immunofluorescence; Table S3: (separate file). Differential gene expression (DEGs) between the UVB-irradiated and unirradiated KC and HaCaT cells.
