# Supplementary material for: Protective Effects of Keratinocyte-Derived GCSF and CCL20 on UVB-Induced Melanocyte Damage
Source: Cells. 2024 Oct 8;13(19):1661. doi: 10.3390/cells13191661 (PMC11475719; doi:10.3390/cells13191661)
Supplement: Supplementary file 1 [file cells-13-01661-s001.zip › Supplementary material_UP_R1.docx]

**Supplementary material**

**Protective Effects of keratinocyte-derived GCSF and CCL20 on UVB-induced melanocyte damage**

Saowanee Jeayeng, Malinee Saelim, Phetthinee Muanjumpon, Pongsakorn Buraphat, Potjanee Kanchanapiboon, Somponnat Sampattavanich*, Uraiwan Panich*

**Correspondence:**

Uraiwan Panich, M.D., Ph.D.

Department of Pharmacology, Faculty of Medicine Siriraj Hospital, Mahidol University, Bangkok 10700, Thailand.

E-mail: uraiwan.pan@mahidol.ac.th

Figure S1. The effects of UVB on melanin content in MC and KC at 12 h after UVB irradiation .

Dose-dependent effects of UVB (31.25, 62.5 and 125 mJ/cm^2^) on melanin content at 12 h post-irradiation in MC (A) and KC (B). The statistical significance of differences between UVB-irradiated MC or KC and UVB-unirradiated MC or KC was evaluated by one-way ANOVA followed by Dunnett's test (***P* < 0.01; ****P* < 0.001 versus UVB-unirradiated MC or KC).


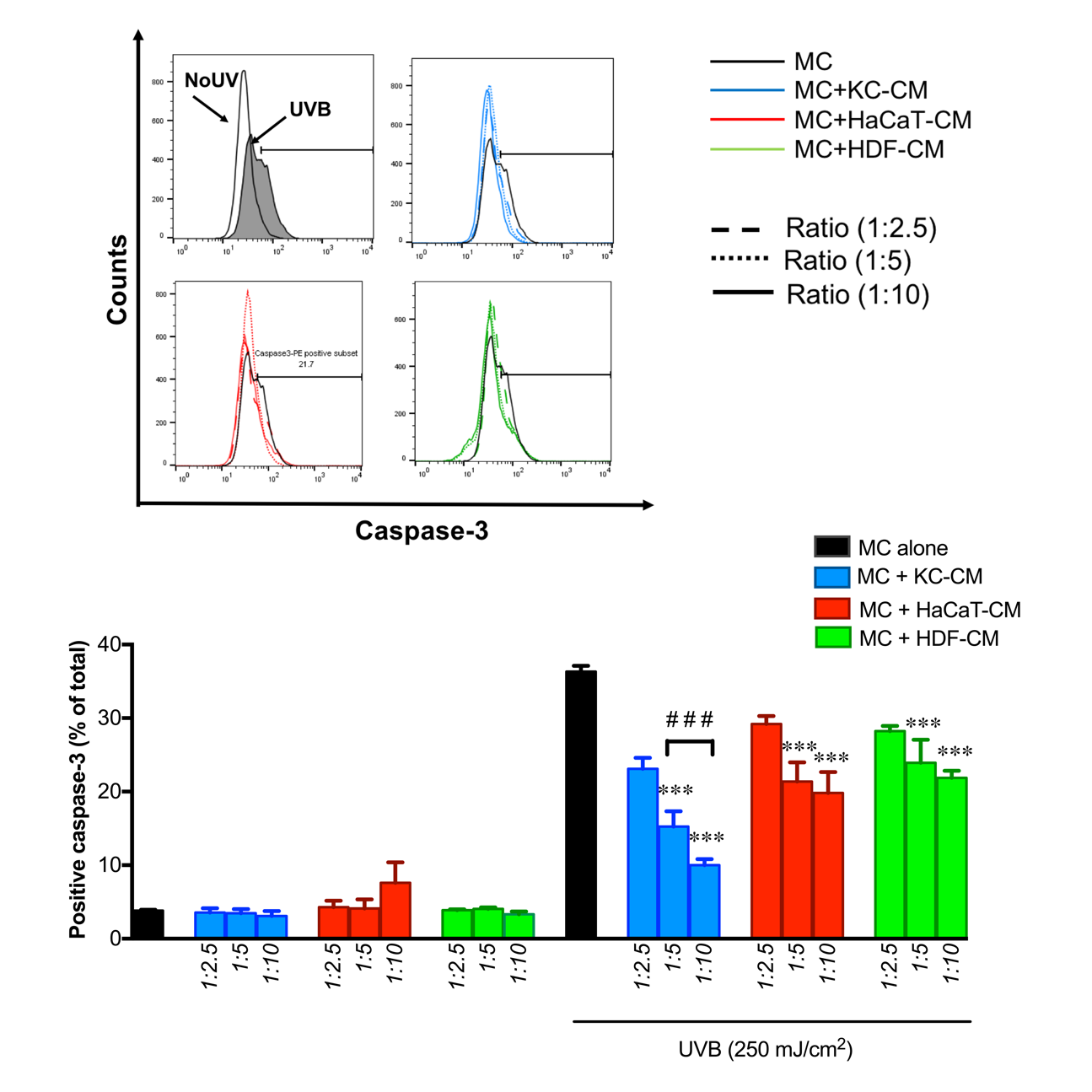
Figure S2. The effects of different cell ratios of KC, HaCaT, HDF on UVB-induced caspase3 activation in MC at 12 h after UVB irradiation .

MC were treated with CM from KC, HaCaT, HDF at three cell ratios (1:2.5, 1:5, 1:10). The statistical significance of differences between UVB-irradiated MC and UVB-irradiated MC+KC-CM, MC+HaCaT-CM, MC+HDF-CM at different cell ratios was evaluated by one-way ANOVA followed by Dunnett's test (****P* < 0.001 versus UVB-irradiated MC). ### *P* < 0.001versus UVB-irradiated MC+KC-CM at cell ratio (1:5).


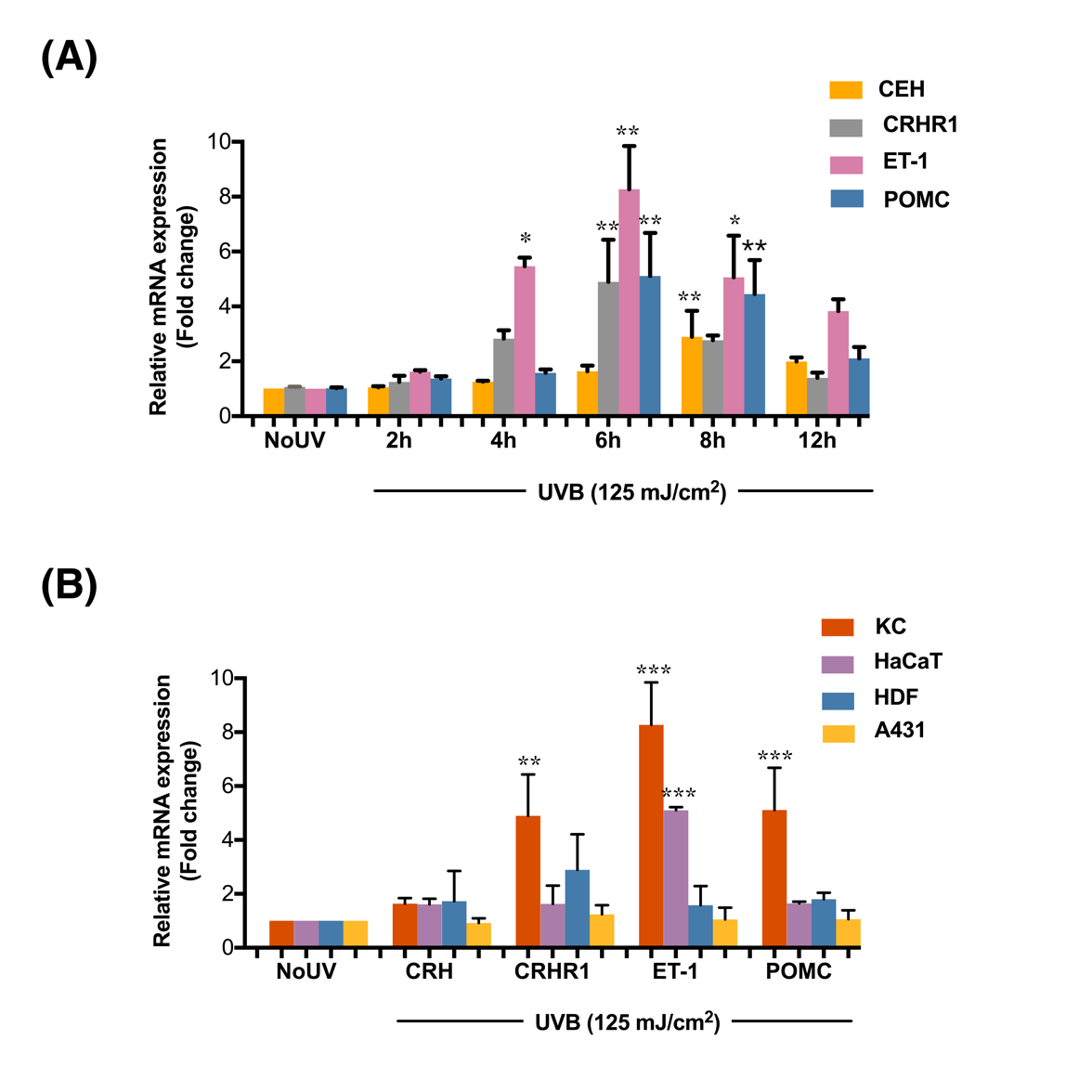


**Figure S3.** **The effects of UVB on genes encoding secreted paracrine factors (CRH, CRH1, ET-1, POMC) expression in KC, HaCaT, HDF, A431 cells.**

Time-dependent effects of UVB (125 mJ/cm^2^) on CRH, CRHR1, ET-1, POMC mRNA expression at 2, 4, 6, 8, 12 h post-irradiation in KC cells (A). The statistical significance of differences was evaluated by one-way ANOVA followed by Dunnett's test. **P* < 0.05; ***P* < 0.01 versus unirradiated KC. The effects of UVB (250 mJ/cm^2^) on mRNA levels of the paracrine factors (ET-1, CRH, CRHR1 and POMC) at 6 h post-irradiation in KC, HaCaT, HDF, A431 cells (B). The statistical significance of differences was evaluated by one-way ANOVA followed by Dunnett's test. (**P* < 0.05; ***P* < 0.01; ****P* < 0.001 versus unirradiated cells.

**
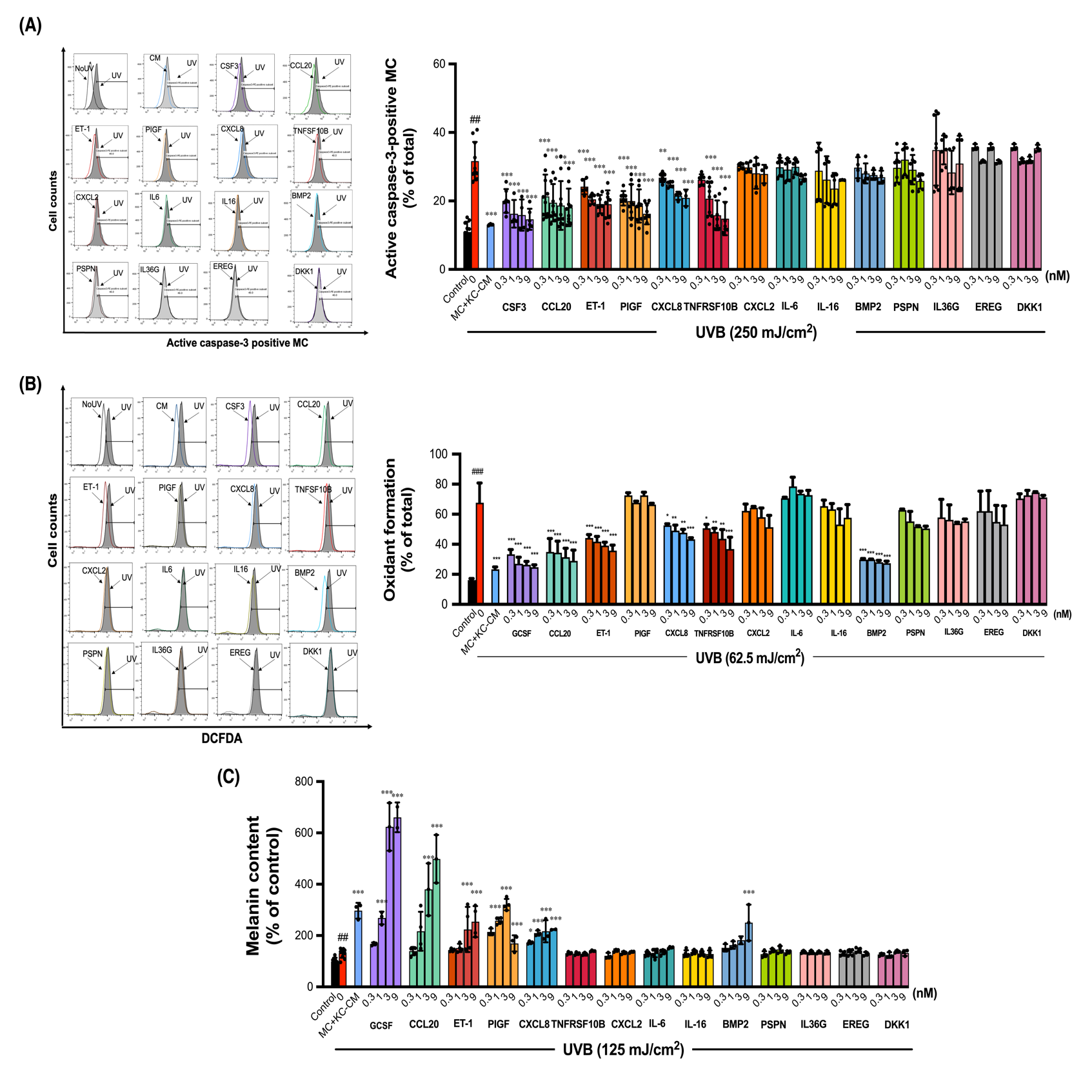
Figure S4. The protective effects of paracrine factors on UVB-induced apoptosis, ROS formation and melanogenesis in MC cells.** The effects of UVB on caspase3 activation (A), ROS formation (B), melanin content (C) in MC pretreated with CM from KC, irradiated with UVB (125 mJ/cm^2^) and 14 recombinant paracrine factors including G-CSF, CCL20, ET-1, PIGF, CXCL8, TNFRSF10B, CXCL2, IL-6, IL-16, BMP2, PSPN, IL36G, EREG and DKK1 at a concentration at dose 0.3, 1, 3, and 9 nM for 2 h. MC were harvested at 12 h after UVB irradiation for determination active caspase-3 staining, melanin content and 1 h after UVB irradiation for determination of ROS formation. Data was expressed as mean ± SD. The statistical significance of differences between UVB-irradiated MC and UVB-irradiated MC+KC-CM, and 14 recombinant paracrine factors was evaluated by one-way ANOVA followed by Dunnett's test (**P* < 0.05; ***P* < 0.01; ****P* < 0.001 versus UVB-irradiated MC).

**
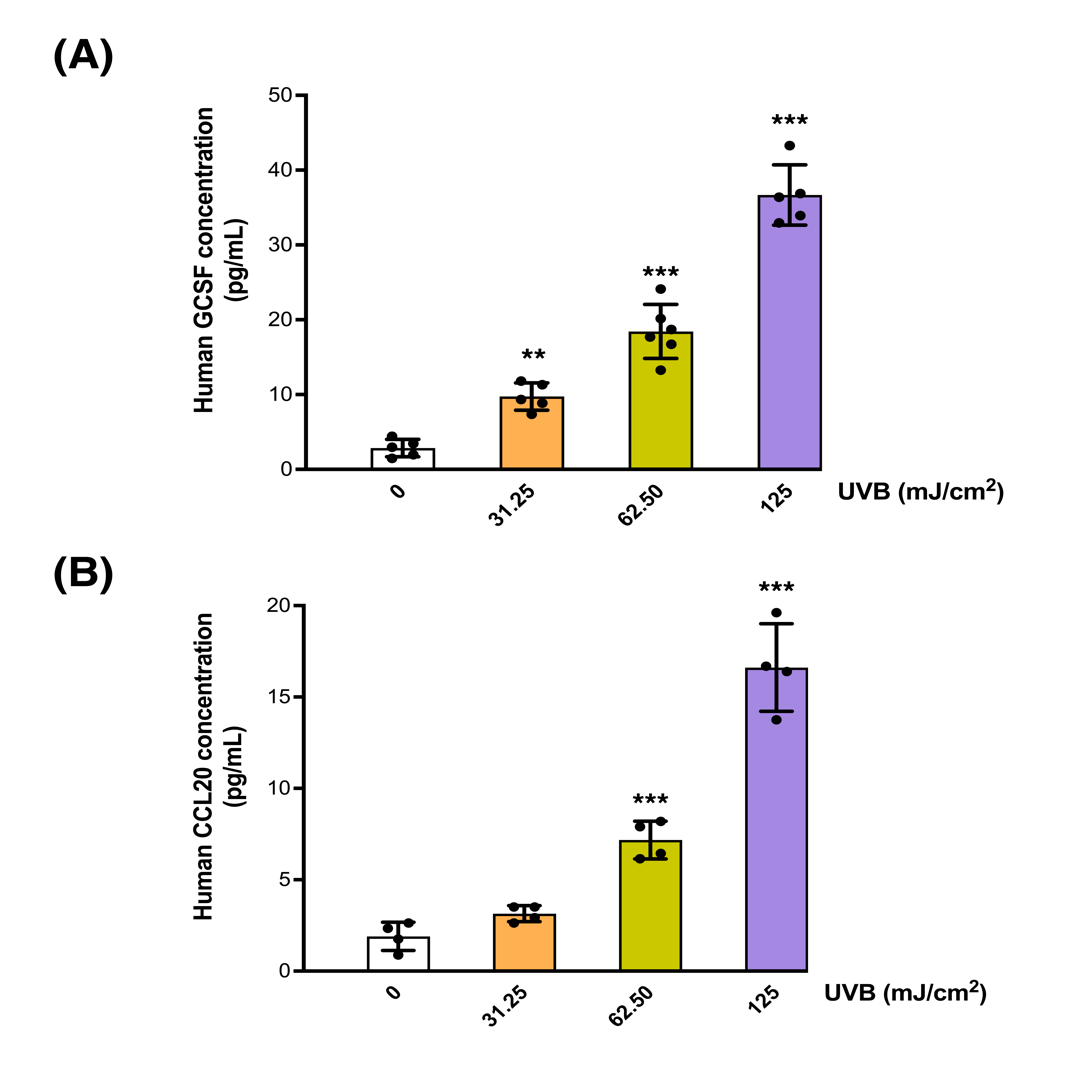

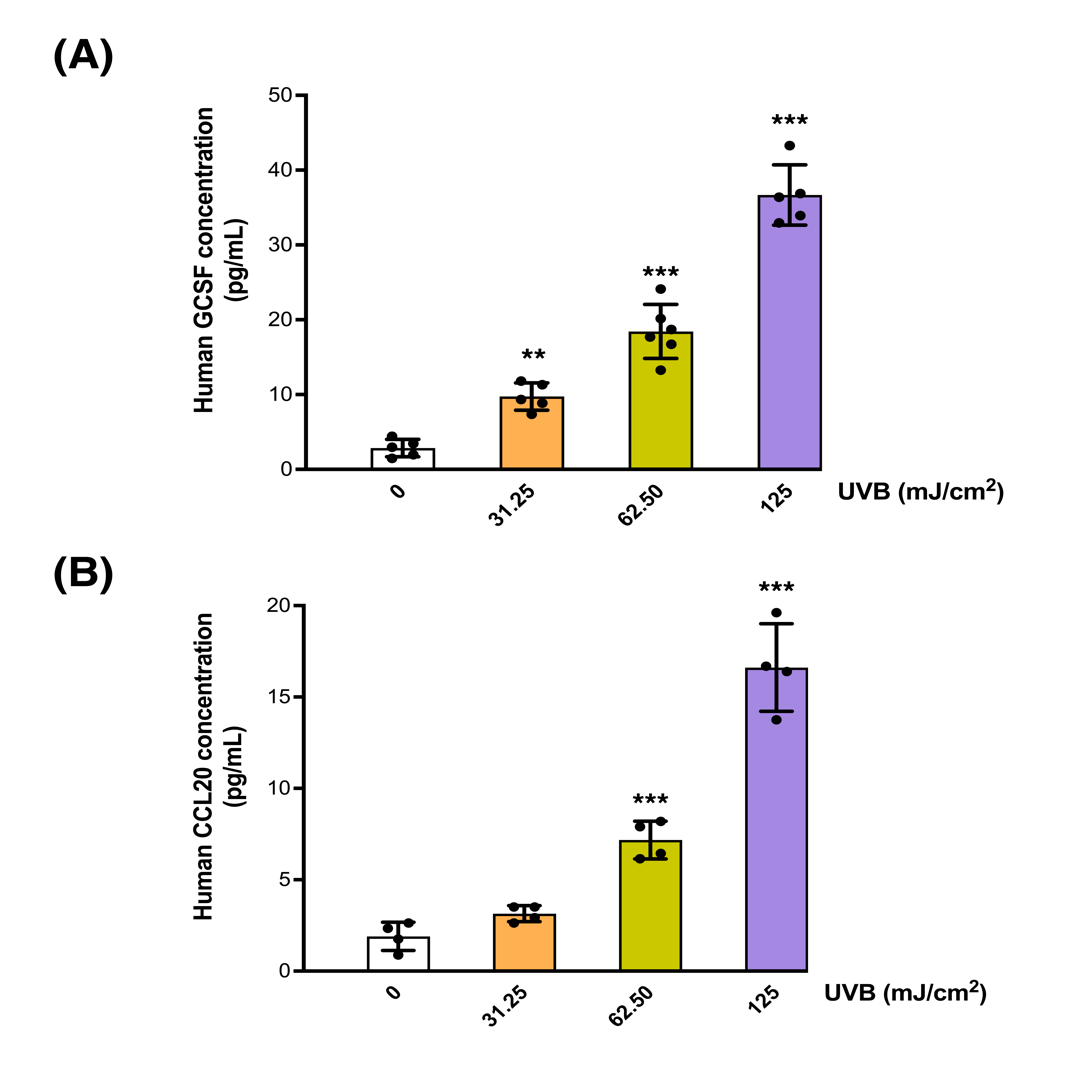
**

Figure S5. The effects of UVB on GCSF and CCL20 levels in KC cells at 12 h after UVB irradiation .

Dose-dependent effects of UVB (31.25, 62.5 and 125 mJ/cm^2^) on GCSF and CCL20 levels in KC cells. At 12 h post-irradiation, CM from KC were collected and then G-CSF (A) and CCL20 (B) concentrations were measured. Data was expressed as mean ± SD. The statistical significance of differences between UVB-irradiated KC and UVB-unirradiated MC was evaluated by one-way ANOVA followed by Dunnett's test (***P* < 0.01; ****P* < 0.001 versus unirradiated control KC).

**
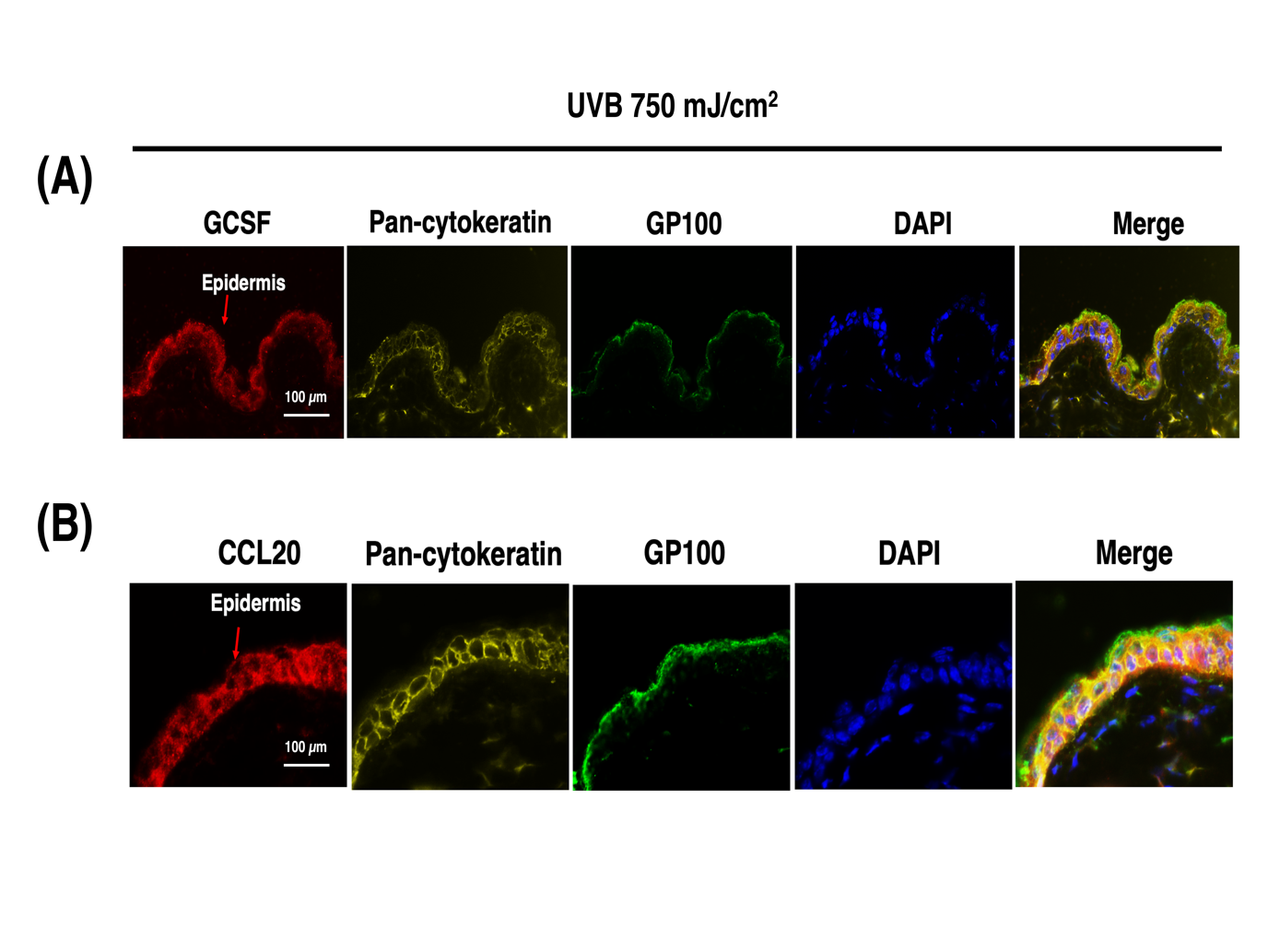
**

**Figure S6.** **The expression of G-CSF and CCL20 protein in mouse skin exposed to UVB irradiation.** The immunofluorescence analysis was performed to determine protein expressions of G-CSF (A), CCL20 (B), epidermal maker pan-cytokeratin, gp100, and nuclear marker DAPI (a and b) at 12 h following UVB (750 mJ/cm^2^) exposure.

**Table S1. Primer sequences**

| No. | Gene | Product Size (bp) | 5' to 3' | Primer sequence |
| --- | --- | --- | --- | --- |
| 1 | *CRH* | 144 | Sence | CTCCGGGAAGTCTTGGAAAT |
|  |  |  | Antisence | GTTGCTGTGAGCTTGCTGTG |
| 2 | *CRHR1* | 100 | Sence | TGGATGTTCATCTGCATTGG |
|  |  |  | Antisence | TGCCAAACCAGCACTTCTC |
| 3 | *ET-1* | 274 | Sence | TCTACTTCTGCCACCTGGAC |
|  |  |  | Antisence | CACTTCTTTCCCAACTTGGAAC |
| 4 | *POMC* | 152 | Sence | AGCCTCAGCCTGCCTGGAA |
|  |  |  | Antisence | CAGCAGGTTGCTTTCCGTGGTG |
| 5 | \| *Tyrosinase* \| \| --- \|  \|  \| \| --- \| | 119 | sense | GCACAGATGAGTACATGGGAGG |
|  |  |  | antisense | CTGATGGCTGTTGTACTCCTCC |
| 6 | *TRP1* | 145 | Sence | \| TCTCAATGGCGAGTGGTCTGTG \| \| --- \|  \|  \| \| --- \| |
|  |  |  | Antisence | CCTGTGGTTCAGGAAGACGTTG |
| 7 | *GAPDH* | 197 | sense | GGTGAAGGTCGGAGTCAACG |
|  |  |  | antisense | TGACAAGCTTCCCGTTCTCAG |

Table S2. Primary and Secondary Antibodies Used for Immunofluorescence

| **No.** | **Antibody** | **Host** | **Dilution** | **Catalog Number** | **Supplier** |
| --- | --- | --- | --- | --- | --- |
| 1 | CCL20 | Rabbit | 1:50 | ab139585 | Abcam, Cambridge, MA, USA |
| 2 | GCSF | Rabbit | 1:50 | ab181053 | Abcam, Cambridge, MA, USA |
| 3 | Tyrosinase | Rabbit | 1:50 | ab180753 | Abcam, Cambridge, MA, USA |
| 4 | p-MITF (pSer180/73) | Rabbit | 1:200 | SAB4503940 | Merck, Darmstadt, Germany |
| 5 | \| p-p53 (Ser15) \| \| --- \|  \|  \| \| --- \| | Rabbit | 1:100 | PA5-104742 | Thermo Fisher Scientific, USA |
| 6 | Pan-cytokeratin (keratinocyte marker) | Rabbit | 1:50 | ab86734 | Abcam, Cambridge, MA, USA |
| 7 | gp100 (melanocyte marker) | Rabbit | 1:50 | ab137078 | Abcam, Cambridge, MA, USA |
| 8 | Alexa Fluor® 647 goat anti-mouse | Goat | 1:200 | ab150115 | Abcam, Cambridge, MA, USA |
| 9 | Alexa Fluor® 488 goat anti-rabbit | Goat | 1:200 | ab150077 | Abcam, Cambridge, MA, USA |
| 10 | Rhodamine Red™-X goat anti-mouse | Goat | 1:200 | R6393 | Thermo Fisher Scientific, USA |

Table S3 (Separate file)

Differential gene expression (DEGs) between the UVB-irradiated and unirradiated KC and HaCaT cells. Both up-regulated, and downregulated genes were identified, giving a total of 1730 genes were identified as being differentially expressed (FDR≤0.05). This table, showing: column 1: gene symbol, column 2: expression changes in KC, column 3: FDR of gene in KC, column 4: expression changes in HaCaT, column 5: FDR of gene in HaCaT.
